# Transcriptomic Analysis Reveals the Effect of Urea on Metabolism of *Nannochloropsis oceanica*

**DOI:** 10.3390/life14070797

**Published:** 2024-06-24

**Authors:** Han Zhu, Zhenli Ye, Zhengru Xu, Li Wei

**Affiliations:** 1 Ministry of Education Key Laboratory for Ecology of Tropical Islands, Key Laboratory of Tropical Animal and Plant Ecology of Hainan Province, College of Life Sciences, Hainan Normal University, Haikou 571158, China; 2 Hainan Observation and Research Station of Dongzhaigang Mangrove Wetland Ecosystem, Haikou 571129, China; 3 International Science and Technology Cooperation Laboratory for Marine Microalgae Ecological Carbon Sinks, Hainan Normal University, Haikou 571158, China; 4 College of Foreign Language, Hainan Normal University, Haikou 571157, China

**Keywords:** adaptation mechanism, urea addition, *Nannochloropsis oceanica*

## Abstract

The eukaryotic microalga *Nannochloropsis oceanica* represents a promising bioresource for the production of biofuels and pharmaceuticals. Urea, a crucial nutrient for the photosynthetic *N. oceanica*, stimulates the accumulation of substances such as lipids, which influence growth and physiology. However, the specific mechanisms by which *N. oceanica* responds and adapts to urea addition remain unknown. High-throughput mRNA sequencing and differential gene expression analysis under control and urea-added conditions revealed significant metabolic changes. This involved the differential expression of 2104 genes, with 1354 being upregulated and 750 downregulated, resulting in the reprogramming of crucial pathways such as carbon and nitrogen metabolism, photosynthesis, and lipid metabolism. The results specifically showed that genes associated with photosynthesis in *N. oceanica* were significantly downregulated, particularly those related to light-harvesting proteins. Interestingly, urea absorption and transport may depend not only on specialized transport channels such as urease but also on alternative transport channels such as the ABC transporter family and the CLC protein family. In addition, urea caused specific changes in carbon and lipid metabolism. Genes associated with the Calvin cycle and carbon concentration mechanisms were significantly upregulated. In lipid metabolism, the expression of genes associated with lipases and polyunsaturated fatty acid synthesis was highly activated. Furthermore, the expression of several genes involved in the tricarboxylic acid cycle and folate metabolism was enhanced, making important contributions to energy supply and the synthesis and modification of genes and macromolecules. Our observations indicate that *N. oceanica* actively and dynamically regulates the redistribution of carbon and nitrogen after urea addition, providing references for further research on the effects of urea on *N. oceanica*.

## 1. Introduction

Nitrogen plays an indispensable role in the growth and development of microalgae, existing in various forms in the marine environment, such as dissolved inorganic nitrogen, dissolved organic nitrogen, and particulate nitrogen. Different nitrogen sources significantly impact the absorption and utilization efficiency of microalgae [1]. Urea, a commonly used nitrogen source in microalgae cultivation, stands out for its ability to provide both organic carbon and nitrogen [2]. Subsequently, urea affects protein synthesis, photosynthesis, cell division and growth, and biomass accumulation. Researchers have conducted extensive studies on the impact of urea concentration on biological growth. For instance, it has been demonstrated that microalgae exhibit a higher rate of absorption and utilization of urea compared to inorganic nitrogen sources [3]. Maintaining an appropriate concentration of organic nitrogen is essential. Organic nitrogen influences the cell cycle, quiescence, and the accumulation of triacylglycerol (TAG) in algal cells. Therefore, urea, which can provide organic nitrogen, is a crucial nitrogen source for algal cells [4]. According to Ali Nawaz Kumbhar’s research, minimal urea supply promotes the highest production of total and neutral lipids in *Chlorella pyrenoidosa* compared to high urea concentrations [5]. However, excessive nitrogen concentrations, particularly ammonium ions, can accumulate inside cells, leading to PSII damage and impacting the photosynthetic process. For example, urea affects lutein production; optimal urea addition increases biomass and lutein content, while excessive urea negatively affects growth and lutein synthesis [6]. Despite urea’s evident impact on the physiological and ecological aspects of microalgae, the precise molecular mechanisms affected remain unclear.

*Nannochloropsis oceanica,* a single-cell green microalga, holds great promise as a biological resource for biofuel and pharmaceutical production. This small green microalga is renowned for its rich composition of photosynthetic pigments, proteins, and polyunsaturated fatty acids, particularly eicosapentaenoic acid (EPA). *N. oceanica* is widely utilized as a model organism, characterized by its rapid growth, excellent photosynthetic efficiency, and high lipid accumulation [7]. Under nitrogen stress, algal growth is limited, usually manifested as a lower biomass growth rate and smaller cell size. This reduction in biomass growth rate and cell size leads to a decrease in chlorophyll content, affecting chlorophyll fluorescence and photosynthetic efficiency. Additionally, algae may adopt various adaptation mechanisms to reduce unnecessary consumption and conserve nitrogen resources [8,9]. *N. oceanica* is highly sensitive to nitrogen and other nutrient elements, and its growth and metabolism require an adequate supply of nitrogen. Adding 2 g/L of urea to the culture medium significantly promotes the growth of *N. oceanica* under conditions of 25 °C and 5% CO_2_ [10]. By using urea at different concentration gradients as a nitrogen source at varying rates, it could increase the protein and fat content, as well as essential amino acids and fatty acid content in *N. oceanica* [11]. A higher cell count in *N. oceanica* was observed, which indicates that the growth of this microalgae can be promoted by urea. However, the impact of urea on lipid content was relatively small [12]. Compared to other nitrogen sources (NaNO_3_, NH_4_NO_3_, NH_4_HCO_3_, (NH_4_)_2_SO_4_), *N. oceanica* cultivated with urea had lower lipid content [13]. However, reducing the urea concentration to 0.2 g/L inhibits cell growth. Under urea-limiting conditions, it may adjust its nitrogen metabolism to adapt to the environment. As mentioned above, urea stress inhibits photosynthesis but is conducive to the formation of oils. Urea can regulate the nitrogen metabolism of microalgae, influencing their lipid accumulation. These different metabolic strategies of *N. oceanica* under urea addition are worth exploring.

In this study, we will explore the effects of different nitrogen nutrition conditions (with and without the addition of urea; NaNO_3_ + urea or NaNO_3_) on the growth of *N. oceanica* through two experimental groups and apply mRNA-seq to reveal the molecular response. Transcriptome analysis revealed that the addition of urea led to differential expression of 2104 genes in the experimental group (NaNO_3_ + urea; U) compared to the control group (NaNO_3_; Ct). The result indicated a significant downregulation of photosynthesis-related genes in microalgae, especially genes related to light-harvesting protein. Interestingly, the absorption and transport of urea may not only rely on a specialized transport mechanism mediated by urease but also alternative transport channels such as the ABC transporter family, and CLC protein family. Adenosine is deaminated to form inosine. Inosine is hydrolyzed to produce hypoxanthine and ribose. Hypoxanthine is further oxidized by xanthine oxidase to form xanthine, which is then oxidized to uric acid [14]. Among these steps, we found that only the expression of purine nucleoside phosphorylase (g9552) was significantly downregulated, with a fold change of 2.48 in *N. oceanica* under Ct vs. U [15]. Additionally, urea induced specific changes in carbon metabolism and lipid metabolism. Genes related to the Calvin cycle and carbon concentration mechanisms were significantly upregulated. In lipid metabolism, genes associated with lipases or polyunsaturated fatty acid enzymes were highly activated. Furthermore, there was enhanced expression in the tricarboxylic acid cycle and folate metabolism, contributing significantly to energy supply and the synthesis and modification of genes or large biomolecules.

## 2. Materials and Methods

### 2.1. Culture Conditions of N. oceanica

*N. oceanica* IMET1 cells were inoculated into modified f/2 liquid medium containing 30 g/L sea salt, 1 g L^−1^ NaNO_3_, 3.65 mg L^−1^ FeCl_3_*6H_2_O, 67 mg L^−1^ NaH_2_PO_4_*H_2_O, 4.37 mg L^−1^ Na_2_EDTA*2H_2_O, trace metal mix (0.36 mg L^−1^ MnCl_2_*4H_2_O, 0.0126 mg L^−1^ NaMoO_4_*2H_2_O, 0.0196 mg L^−1^, CuSO_4_*5H_2_O, 0.044 mg L^−1^ ZnSO_4_*7H_2_O, and 0.01mg L^−1^ CoCl_2_), and vitamin mix (2.5 µg L^−1^ biotin, 2.5 µg L^−1^ VB_12_, and 0.5 µg L^−1^ thiamine HCl), and 100mM Tris-HCl (pH = 7.8) [16]. Microalgal cells were cultivated in f/2 medium under continuous irradiation (light intensity: 50 ± 5 μmol m^−2^ s^−1^) at 25 °C in a 1 L column reactor (inner diameter: 5 cm). During the logarithmic growth phase (OD_750_ = 3.0), cells were harvested via 5000 rpm centrifugation. Subsequently, the microalgae cells were washed three times with nitrogen-free sterile seawater. One batch was cultured in a medium supplemented with sodium nitrate (as a control group: Ct) at an inoculation density of OD_750_ = 0.46. Another batch was cultured in a medium supplemented with both sodium nitrate and urea (the final urea density of urea addition 2g/L; as an experimental group: U) with OD_750_ = 0.46, and physiological parameters were measured. After transfer to two fresh-medium conditions at 72 h, cell aliquots from the urea addition samples (U) and control group (Ct) were collected for RNA isolation, with three replicates. Fv/Fm (the variable/maximum fluorescence ratio), the maximum photochemical quantum yield of PSII reaction centers, represents the minimum fluorescence yield when PSII reaction centers are fully open and reflects the photosynthetic light energy conversion efficiency. This parameter was measured using AquaPen AP110-C. To quantify the amounts of carbohydrate, protein, and lipid, *N. oceanica* cells were harvested after 14 days of cultivation by centrifugation at 4000 rpm for 5 min under both Ct and U conditions. The harvested cells were then lyophilized for two days to obtain dried algal powder. The extraction and quantification of lipids, carbohydrates, and proteins from the microalgal biomass were carried out according to our previously published protocols. The physiological data for each time point are presented as mean values with standard deviations. One-way ANOVA was applied to compare the growth rates across different conditions and time points.

### 2.2. Total RNA Extraction

RNA extraction from microalgal cells involved the use of TRIzol^®^ Re-agent and chloroform following Li’s protocols (Invitrogen, Carlsbad, CA, USA). Genomic DNA elimination was achieved using DNase I (TaKara) [17]. Subsequently, the 2100 Bioanalyzer (Agilent Technologies, Inc., Santa Clara, CA, USA) assessed the integrity and purity of the total RNA, while quantification was performed with the ND-2000 (NanoDrop Thermo Scientific, Wilmington, DE, USA) and Qubit 3.0 (Life Technologies, Carlsbad, CA, USA). Only high-quality RNA samples meeting the criteria of OD260/280 = 1.8~2.2 and OD260/230 ≥ 2.0 were selected for the construction of the sequencing library.

### 2.3. Illumina Novaseq 6000 Sequencing and mRNA Sequencing Library Preparation

RNA purification, reverse transcription, library construction, and sequencing were conducted at Shanghai Majorbio Bio-pharm Biotechnology Co., Ltd. (Shanghai, China), following the guidelines of the Illumina manufacturer (San Diego, CA, USA). The creation of the four mRNA-seq libraries utilized the Illumina TruSeq^TM^ RNA Sample Preparation Kit (San Diego, CA, USA). Initial steps involved total RNA purification and isolation using oligo-dT-attached magnetic beads, followed by RNA fragmentation with a fragmentation buffer. Subsequently, short RNA fragments served as templates for double-stranded cDNA synthesis using the SuperScript Double-Stranded cDNA Synthesis Kit (Invitrogen, Carlsbad, CA, USA) with random hexamer primers. The synthesized double-stranded cDNA underwent end-repair, phosphorylation, and “A” base addition as per Illumina’s library construction protocol. Target fragments of 200–300 bp were selected, and libraries were run on 2% Low Range Ultra Agarose post PCR-amplification using Phusion DNA Polymerase (New England Biolabs, Boston, MA, USA) for fifteen cycles. After quantification, the four RNA sequencing libraries underwent sequencing in a single lane on an Illumina NovaSeq 6000 platform (Illumina, San Diego, CA, USA) for 2 × 150 bp paired-end reads.

### 2.4. Transcriptome Assembly and Functional Annotation

The raw paired-end reads of mRNA-seq were trimmed and data quality was controlled using software of SeqPrep and Sickle with default parameters. Then clean data from the samples (Ct) were employed to perform de novo assembly with Trinity [18]. All the assembled transcripts were searched against the protein nonredundant (NR) of NCBI, String, and KEGG databases using BLASTX to identify the proteins that had the highest sequence similarity with the given transcripts to retrieve their function annotations and a typical cut-off E-values less than 1.0 × 10^−5^ was set. BLAST2GO [19] program was used to obtain GO annotations of unique assembled transcripts for describing biological processes, molecular functions, and cellular components. Metabolic pathway analysis was performed using the KEGG (Kyoto Encyclopedia of Genes and Genomes) [20]. Raw sequencing data are assessed for quality using FastQC and then subjected to quality trimming using Trimmomatic, resulting in relatively accurate and valid data. Clean data are assembled into transcripts de novo using Trinity [21]. The effective data from the samples are subjected to mixed splicing assembly, resulting in transcripts, and the sequence length and GC content of the assembled sequences are counted. The assembled transcripts are used as a reference sequence, and the sequencing data are compared and analyzed against them to filter sequencing sequences that can be mapped to the reference sequence.

### 2.5. Differential Expression Analysis and Functional Enrichment Analysis

Identification of differentially expressed genes (DEGs) between two distinct samples involved analyzing the expression levels of individual transcripts using the Fragments Per Kilobase of Exon Per Million Mapped Reads (FPKM) method. Quantification of gene and isoform abundances was performed using the RSEM tool [22]. The R statistical package software EdgeR (Empirical Analysis of Digital Gene Expression in R, facilitated the differential expression analysis [23]. Furthermore, functional enrichment analysis, encompassing Gene Ontology (GO) and Kyoto Encyclopedia of Genes and Genomes (KEGG), was conducted. This aimed to identify DEGs significantly enriched in GO terms and metabolic pathways at a Bonferroni-corrected *p*-value ≤ 0.05 when compared with the entire transcriptome background. The GO functional enrichment and KEGG pathway analysis were carried out using two software tools, namely Goatools and KOBAS [24].

Typically, the Pearson correlation coefficient is used as an evaluation metric for replicative correlation [25], and the closer the coefficient value is to 1, the higher the similarity of expression characteristics between samples.

The volcano plot is the most commonly used method to display the results of differential gene expression analysis. It uses a *t*-test to analyze genes with significant differential expression between two samples, with log_2_ (fold change) on the x-axis and the negative logarithm −log10 (q-value) of the *p*-value from the *t*-test on the y-axis. The volcano plot includes two important metrics: fold change and adjusted q-value. Each point on the plot represents a gene, with colors used to differentiate whether genes are differentially expressed. In the control group, there are 212 unique genes, accounting for 2.24% of the total genes. In the experimental group, there are 197 unique genes, making up 2.08% of the total genes. There are 9051 genes shared between the two groups, representing 95.68% of the total genes.

### 2.6. qPCR Experiment for Validating mRNA-seq

To minimize bias stemming from different biological replicates, six genes were selected for RT-qPCR analysis. The same samples used in mRNA-seq experiment were employed for RT-qPCR. The M-MLV Reverse Transcription Kit (Promega, Madison, WI, USA) was used to synthesize cDNA following the manufacturer’s instructions. Gene-specific primers (Table S1) were designed using Primer5 software (Primer Premier 5.0). A 20 μL reaction mixture was prepared using the SYBR Green qPCR Kit Master Mix (Roche, South San Francisco, CA, USA) and run on the real-time PCR LifeCycle480 system (Roche, USA) according to the manufacturer’s protocol. The cycle threshold value (CT) and differential expression fold change were calculated based on the 2^−ΔΔCT^ method with the actin gene of *N. oceanica* IMET1 as the endogenous reference. Each sample was run in triplicate to confirm the reproducibility of the results.

## 3. Results and Discussion

### 3.1. Physiological Changes after Urea Addition

**Figure 1:**
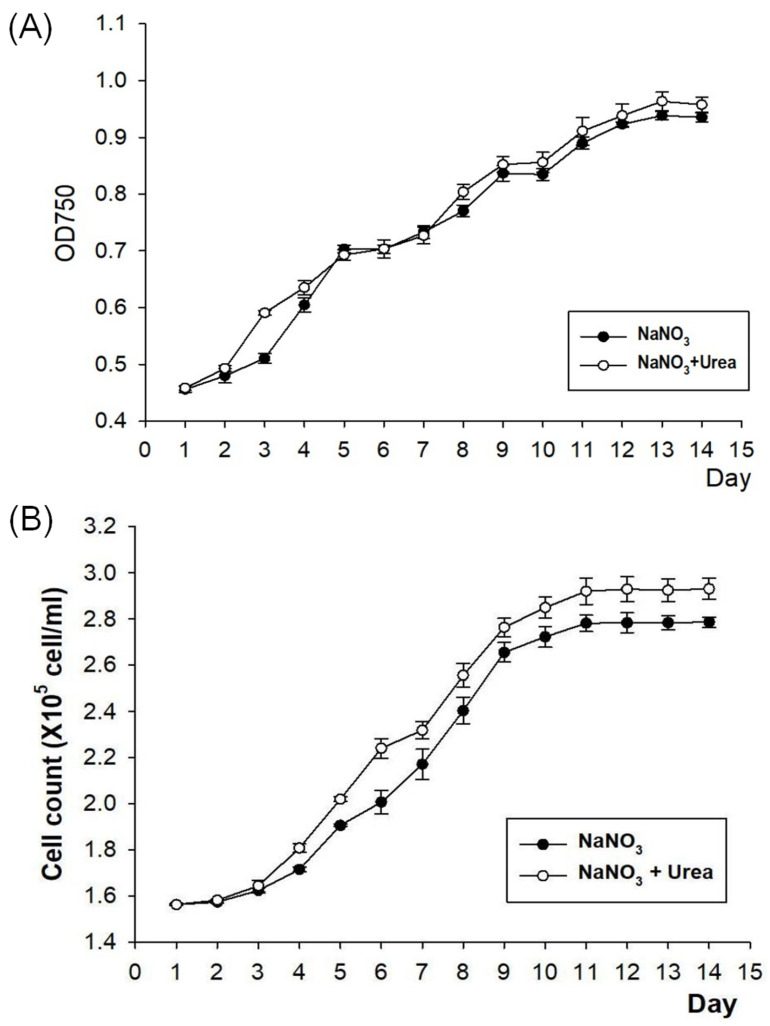
Growth curves of *N. oceanica* under two different conditions. Optical density (**A**) and cell count (**B**) of *N. oceanica* under two different conditions. Values shown as mean ± standard deviation (SD) of three biological replicates.

To survey the impact of urea addition on growth, the physiological responses of *N. oceanica* IMET1 were tracked under two contrasting culture conditions: NaNO_3_ (Ct: control) and NaNO_3_ + urea (U: experimental group, with a final urea concentration of 2 g/L). In this experiment, the growth of microalgae was assessed by measuring the optical density (OD) of the algal culture using a spectrophotometer. After a 14-day cultivation period, we found that the growth of the urea-added group was significantly faster than that of the control group on the third day. However, there were no significant differences in the growth of microalgae between the control group and the experimental group from the fifth to the eighth day (Figure 1A). After the eighth day, the growth of the experimental group displayed a slight increase compared to the control group (Figure 1B). However, the urea addition did not result in a statistically significant increase in growth. Thus, we conclude that urea treatment did not negatively affect the growth of *N. oceanica* cells.

### 3.2. Molecular-Level Response of N. oceanica to Urea 

#### 3.2.1. Transcriptome Data of *N. oceanica* from Illumina Sequencing

TPM (Transcripts Per Million) is a common method for estimating gene expression levels, which considers the true expression level of genes, gene length, and sequencing depth’s impact on reads counting [26]. The correlation of gene expression between samples is an important indicator for assessing the reliability of experiments and the rationality of sample selection [27]. In addition, qPCR was performed to validate mRNA-seq data, and the results demonstrated the quality of mRNA-seq was reliable for further analysis (Figure S1).

**Figure 2:**
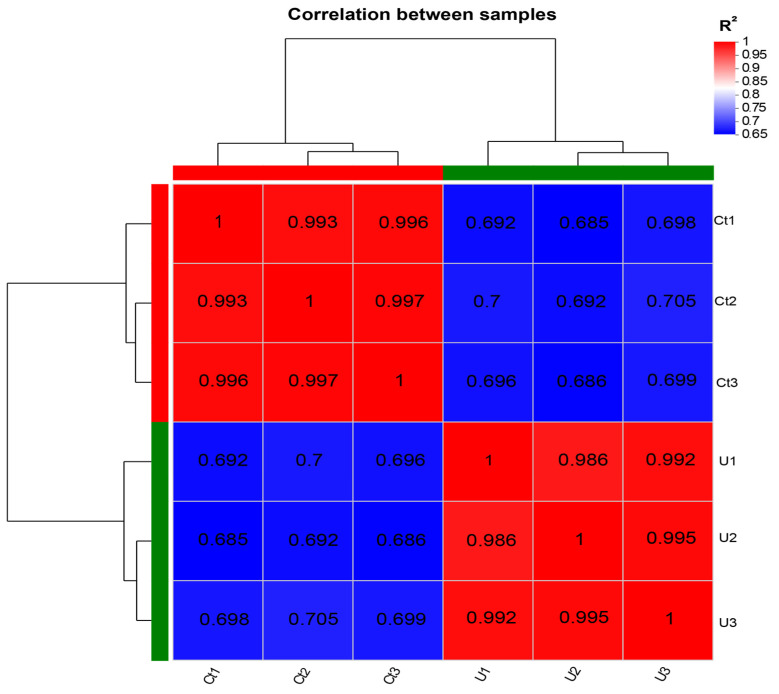
Primary analysis of mRNA-seq data in *N. oceanica*. Sample correlation analysis heatmap. Note: Red indicates strong correlation, and blue indicates weak correlation.

Based on the correlation analysis Heatmap chart of the six samples, the correlation among the three replicates in the control group (Ct1, Ct2, Ct3) ranges from 0.993 to 1, while in the experimental group (U1, U2, U3), the correlation among the three replicates falls between 0.986 and 1. The strong correlation within each group confirms the reliability of the data quality and indicates that it can be used for further analysis. To further elaborate on these findings, the high correlation values suggest a high degree of consistency and reproducibility among the biological replicates within each group. This level of reproducibility is crucial for ensuring that observed differences between control and experimental conditions are due to the experimental treatment rather than variability in sample processing or measurement errors (Figure 2).

#### 3.2.2. Sample Correlation Analysis Inter-Sample Venn Analysis and Differentially Gene Expression Analysis by the Volcano Plot

**Figure 3:**
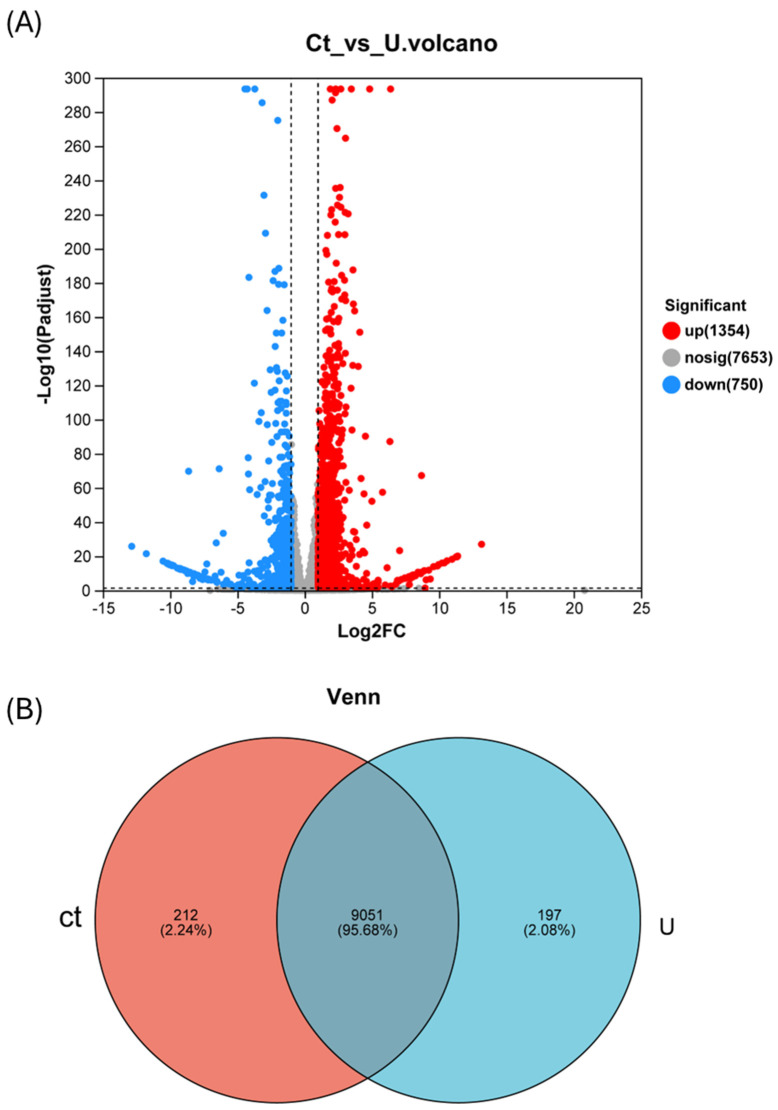
Venn diagram between samples and analysis of differentially expressed genes. (**A**) Volcano plot illustrating the differential expression pattern of genes between Ct and U conditions. Red dots, blue dots, and gray dots represent upregulated DEGs, downregulated DEGs, and genes with no significant change (Ct), respectively. (**B**) The red portion represents unique genes from the group without urea addition, the blue portion represents unique genes from the group with urea addition and the region at the intersection of the two parts represents shared genes. Quantification of differentially expressed genes in *N. oceanica* under U (NaNO_3_ + urea) and Ct (NaNO_3_) cultivation conditions (at least a 2-fold change under urea addition).

The upregulated genes exhibited a higher fold change in expression compared to the downregulated genes, and the differential expression of upregulated genes was more pronounced than that of downregulated genes. Differentially expressed genes were identified using DESeq in R, with filtering criteria set to q-value < 0.05 and |fold change| > 2 (Figure 3A).

The differential gene expression analysis between the urea-added group and the urea-free group of the marine microalgae *Nannochloropsis oceanica* revealed a total of 2104 differentially expressed genes. Among these, 1354 genes were upregulated, while 750 genes were downregulated (Figure 3B).

#### 3.2.3. Functional Enrichment of Differential Expressed Gene by GO and KEGG

**Figure 4:**
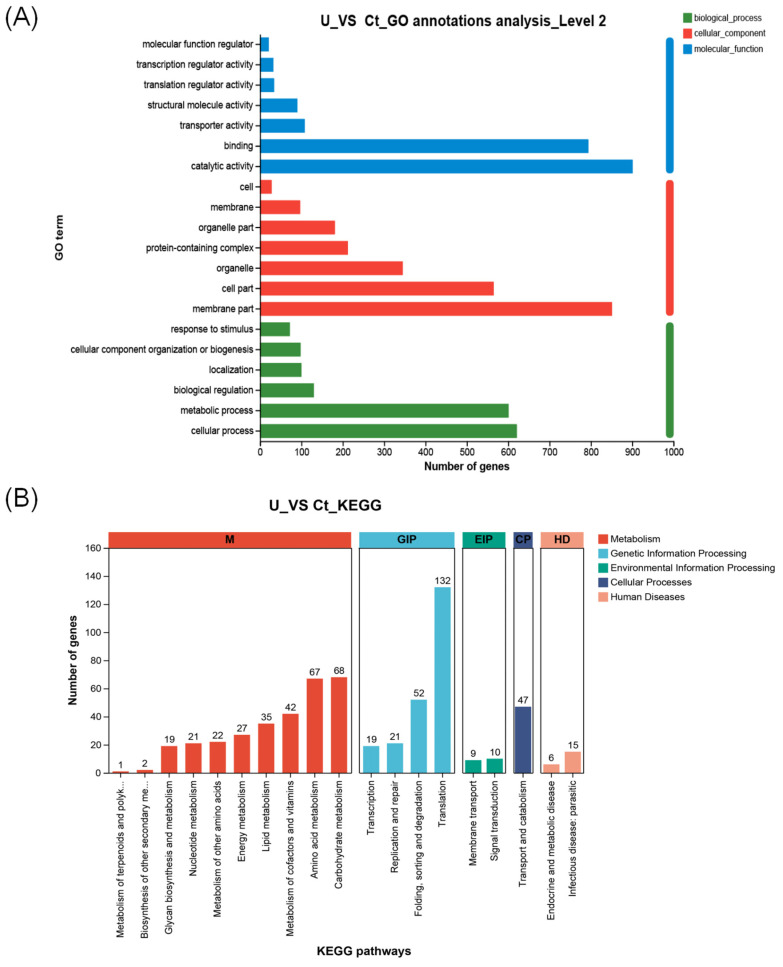
Functional enrichments and classifications of differentially expressed genes in *N. oceanica.* (**A**) The *N. oceanica* genes were systematically categorized into biological process (BP), cellular component (CC), and molecular function (MF) subclasses through Gene Ontology (GO) analysis. The distribution of genes in each class was visualized using a histogram. (**B**) Additionally, a pathway enrichment analysis of DEGs was conducted using the KEGG.

Gene ontology classification annotation was performed on the obtained genes, as shown in Figure 3. From the graph, we can observe that the majority of genes are annotated to the cellular component category, while the differences in genes annotated to biological processes and molecular functions are less pronounced. Within the biological process category, there are a total of six enriched functions, including “response to stimulus”, “cellular component organization or biogenesis”, “localization”, “biological regulation”, “metabolic process” and “cellular process”. Among these, “metabolic process” and “cellular process” show significant enrichment, each category encompassing approximately 600 genes. In the cellular component category, the affected parts include “cell”, “membrane”, “organelle part”, “protein-containing complex”, “organellecell part” and “membrane part”. Notably, the “single-cell part” and “membrane part” exhibit the most significant differential gene enrichment, with approximately 600 and 850 genes enriched, respectively. In the molecular function category, the functions associated with differentially expressed genes include “molecular regulator activity”, “transcription regulator activity”, “translation regulator activity”, “structural molecule activity”, “transporter activity”, “binding” and “catalytic activity”. Differential gene enrichment is particularly pronounced in the “binding” and “catalytic activity’ functions”, with approximately 800 and 900 genes enriched in these categories, respectively (Figure 4A).

After performing KEGG annotation on differentially expressed genes and classifying KEGG metabolic pathways based on the connections between KEGG and pathways, they are mainly divided into five categories: environment information processing (this category includes two pathways: membrane transport and signal transduction), human diseases (includes two pathways: endocrine and metabolic diseases and infectious diseases: parasitic), cellular processes (under this category, there are pathways related to the metabolism of terpenoids and polyketides), metabolism (this category encompasses ten pathways: biosynthesis of other secondary metabolites, glycan biosynthesis and metabolism, nucleotide metabolism, metabolism of other amino acids, metabolism of cofactors and vitamins, lipid metabolism, transport and catabolism, energy metabolism, carbohydrate metabolism, and amino acid metabolism), genetic information processing (four pathways: transcription, folding, sorting, and degradation, replication and repair, and translation). In terms of metabolic functions, the highest enrichment of differentially expressed genes is observed, especially in carbohydrate metabolism pathways, where 68 genes are enriched. Most of these genes involved in controlling photosynthetic carbon fixation are upregulated. Additionally, differential gene enrichment in lipid metabolism pathways is also noticeable, with 35 genes enriched (Figure 4B).

### 3.3. Nitrogen Metabolism Affected by Urea Addition in N. oceanica

**Figure 5:**
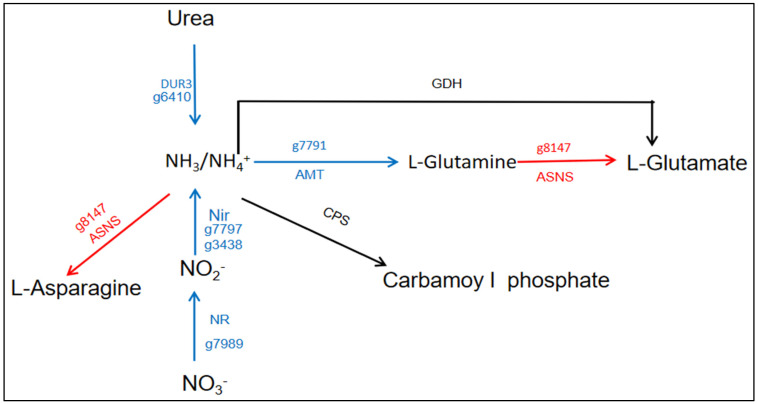
Nitrogen metabolism-related genes differential expression analysis in *N. oceanica* under urea addition conditions. Brief construction of the nitrogen assimilation pathway and transcriptional level changes in key genes marked with arrows. The different colored arrows represent the transcriptional changes in the gene, the “red arrows” and the “blue arrows” represent the upregulation and downregulation of the urea addition condition, respectively.

To study the impact of urea addition on nitrogen metabolism, we analyzed the main pathways of nitrogen assimilation. Upon annotating the transcriptome three genes (urea/Na+ high-affinity symporter, nitrate high-affinity transporter, and ammonium transporter) related to nitrogen transporter exhibited significant downregulation (Figure 5 and Table 1). Among them, urea/Na+ high-affinity symporter (g6410) involved in the urea transport process, downregulated by a fold change of 13.33 under Ct vs. U. Under urea-added cultivation conditions, *N. oceanica* tends to preferentially utilize NH_4_^+^ [28]. However, the transcriptional expression of the ammonium transporter gene differs significantly from the expected pattern. This preference may be mediated through a low-affinity ammonium transport protein system (LATS) [29]. In addition, nitrate high-affinity transporter (g7989) is responsible for nitrate absorption and transport, showing a 9.36-fold downregulation in the experimental group. Ammonium transporter (AMT: g7791) is a type of NH_4_^+^ transport protein responsible for the transport of NH_4_^+^ within organisms. Under Ct vs. U, the expression of this ammonium transport protein was downregulated by 21.74-fold. The ammonium transporter gene possesses a conserved C-terminal regulatory domain and a highly protein homologous structure. It can form trimers by binding with other related monomeric transport proteins to facilitate the transport of NH_4_^+^. Additionally, studies indicate that the conserved C-terminal regulatory domain of the ammonium transporter protein is regulated by phosphorylation at two threonine residues, T460 and T472 [30,31]. When the external NH_4_^+^ concentration is low, the phosphorylation state of the serine residues at the C-terminal region determines whether dephosphorylation occurs, thereby influencing NH_4_^+^ absorption or inhibition [32,33].

Nitroreductase-like protein (g7797) is involved in the reduction of nitrite to ammonia or other nitrogen compounds, and its expression level has decreased by 28.57-fold (Figure 5 and Table 1). Ferredoxin nitrite reductase (g3438) is also involved in the nitrate reduction metabolic pathway, converting nitrate into nitrite. However, based on the provided data, its expression level is downregulated by 17.54-fold (Figure 5 and Table 1). This phenomenon indicates the inhibition of the nitrogen absorption pathway for nitrate. In general, urea is absorbed by algae through two pathways: urease or ureamide hydrolase (DUR) degradation to produce ammonia, which is subsequently converted to other forms of nitrogen via the GS cycle [34]. It has been reported that urease activity in algal cells is regulated by nitrogen species in the culture environment [35]. Urea and NO_3_^−^ can enhance it, and NH_4_^+^ can inhibit it. Interestingly, it was found that the expression of the *Dur3* gene under different nitrogen source conditions was also affected by nitrogen source type, and the addition of urea and NH_4_^+^ inhibited the expression of the *Dur3* gene [36]. In addition, some prokaryotes, such as *Cyanobacteria*, can also be driven to take up urea by ABC binding box (ATP-binding protein) transporters [35]. Research has shown that the expression of various nitrogen transport proteins and auxiliary proteins in the experimental group is significantly downregulated. This observed downregulation occurs in *Arabidopsis*, wheat seedlings, and various algae [37,38]. However, we note that the transcriptional levels of some possible low-affinity transport channels are significantly upregulated. This suggests that algae may employ various nitrogen transfer mechanisms to accomplish the absorption of nitrogen sources [3]. The expression of some ABC protein families is significantly upregulated (Dataset S1). This may indicate the involvement of ABC protein families in the transport of urea in *N. oceanica*. These protein families typically involve ATP binding and hydrolysis to facilitate the transmembrane transport of substances [39,40]. In addition, Chloride Channel Protein 7 (g3611) is a member of the CLC protein family, and it exhibits a significant upregulation at the mRNA level with a fold change of 3.67 (Dataset S1). CLC transport proteins belong to the nitrate transporter protein family. Previously, it has been discovered that CLCa and chloroplast-localized CLCe cooperatively participate in the assimilation process of nitrate nitrogen [41]. In *N. oceanica*, Chloride Channel Protein 7 (g3611) might perform the same function.

At the same time, we found that the GS/GOGAT cycle is highly activated, and the genes for aspartate synthetase and its associated amino acids (such as serine, tryptophan, and nucleotides) are significantly upregulated at the transcriptional level. This indicates that nitrogen utilization and transfer as well as amino acid anabolism are active under urea addition. In *Arabidopsis*, urea-derived ammonia is converted into glutamine, and then further synthesized into glutamate by the GS/GOGAT cycle. This indicates that the addition of urea promotes the activity of the GS/GOGAT cycle [42]. There is significant activity in both ammonia assimilation and ammonia dissimilation. For instance, in the direction of ammonia assimilation, the expression of gene delta-1-pyrroline-5-carboxylate synthetase (g10184) is upregulated by 6.46-fold, gene kynureninase (g9735) is upregulated by 177.16-fold, and gene asparagine synthase (g8147) is upregulated by 63.69-fold (Table 1). These genes are involved in the synthesis and metabolism of amino acids. Regarding ammonia dissimilation, the gene (g615) encodes uricase, which is involved in uric acid metabolism, while the function of gene putative urate catabolism protein (g6250) is suspected to be related to uric acid breakdown metabolism (Table 1). the genes encoded urate oxidase and putative *urate catabolism protein* each show an upregulation of about 2-fold at the mRNA level. As for the urea cycle, although there were no detected differential expressions in the genes encoding Ornithine Transcarbamylase (OTC) and Argininosuccinate Lyase (ASL), the gene expression of argininosuccinate synthase (As: g9579) showed a substantial upregulation (2.12-fold) (Dataset S1). Argininosuccinate synthase plays a crucial role in the synthesis of argininosuccinate from citrulline and aspartate, a key step in the conversion of arginine to urea in the urea cycle. This upregulation may indicate a cellular response to increased demand for argininosuccinate or enhanced urea cycle activity. These indicate that in response to additional urea supply, *N. oceanica* adopted a series of regulatory reactions to maintain the balance of nitrogen metabolism. These regulatory reactions include that conventional nitrogen transporters are inhibited and may turn to low-affinity transport channels to enhance nitrogen utilization and transfer through amino acid synthesis and metabolism. In the physiological data measurements, the total amount of protein measured in the experimental group was higher than that in the control group, which provides corroborative evidence (Figure S2).

### 3.4. Change in Photosynthesis in Response to Urea Addition

In order to explore whether urea addition has an effect on photosynthesis, we conducted a detailed analysis of transcripts related to the photosystem I and II, and chlorophyll biosynthesis genes in *N. oceanica*. The presence of urea significantly downregulates the expression of genes associated with both the light-harvesting complex proteins (LHC) and photosystem proteins. Notably, most *LHC* genes (including g240, g5628, g3077, g6113, g5629, g9713, g903) uniquely showed a 2.0~2.5-fold downregulation at the transcript level (Table 2). Additionally, other genes encoded (cytochrome c biogenesis protein, thiol reduction transmembrane region) also displayed significant downregulation, which indicates potential inhibition of photosynthetic activity occurred in *N. oceanica*. In fact, we measured the photosynthetic parameter Fv/Fm. The results showed that, over time, the Fv/Fm values of the experimental group with urea addition gradually decreased. This confirms that the addition of urea may have an inhibitory effect on *N. oceanica* photosynthetic efficiency (Figure S3). In a previous study, ammonium limitation in algal cultivation may impair photosynthesis by damaging the photosystem or uncoupling photophosphorylation with electron transport [43]. Furthermore, the addition of urea in *Gracilariopsis lemaneiformis* led to a decrease in photosynthetic pigment proteins, potentially prioritizing the degradation of these proteins during urea substitution, serving as a strategy for rapid restructuring and balancing nitrogen metabolism in algal cells [44]. On the other hand, reducing pigment content in plants can improve water and nitrogen utilization efficiency [45], supported by studies showing an increase in biomass in photosynthetic organisms through reduced pigment content [46,47]. Additionally, we observed an upregulation of the expression of cytochrome b6-f complex iron–sulfur subunit (g9470) by 2.42-fold at the mRNA level (Table 2). This complex is crucial in the chloroplast, participating in electron transport and proton pumping during photosynthesis [48]. The upregulation of iron–sulfur assembly-like protein (g4344) and ribulose-phosphate 3-epimerase (g5017) suggests adaptations to different growth conditions and photosynthetic requirements under urea + NaNO_3_ or NaNO_3_ (Table 2). These observations indicate that urea likely hydrolyzed to produce CO_2_ and participates in the carbon concentrating mechanism (CCM) as a new carbon source, optimizing nitrogen supply and prompting *N. oceanica* to adjust its photosynthetic carbon fixation strategy.

### 3.5. Change in Carbon Fixation and Central Carbon Metabolism in Response to Urea Addition

**Figure 6:**
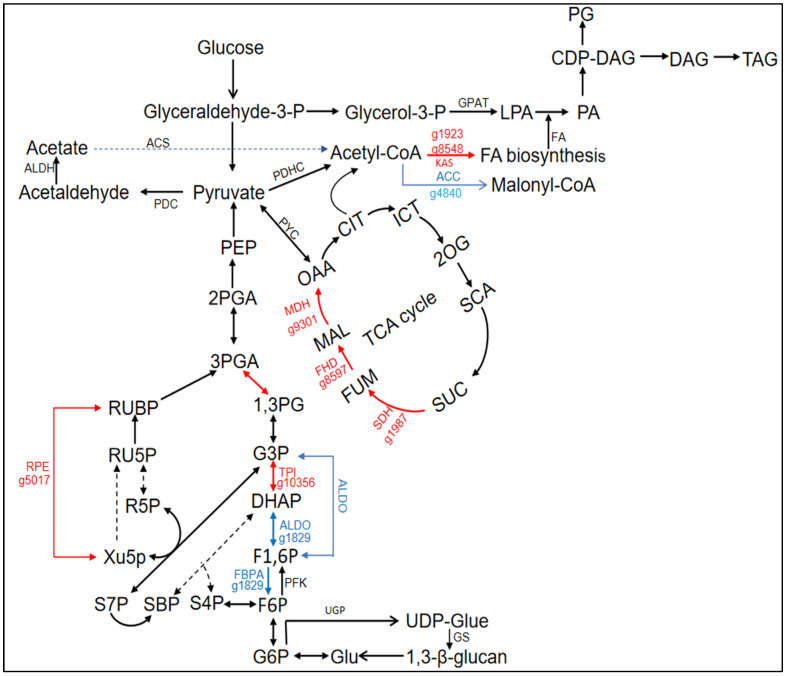
Differential expression analysis of genes related to lipid metabolism and carbon metabolism in *N. oceanica* under urea addition. The carbon metabolism pathway and lipid metabolism pathway were briefly constructed. The different colored arrows represent the transcriptional changes in the gene, the “red arrows” and the “blue arrows” represent the upregulation and downregulation under Ct vs. U, respectively.

To survey urea addition on the CCM (carbon concentrating mechanism) and photosynthetic carbon fixation in *N. oceanica*, we first explored transcriptional changes in various pathways, consisting of glycolysis, gluconeogenesis, the carbon concentrating mechanism, and Calvin–Benson cycle (Figure 6 and Table 1).

Firstly, as for CCM, one carbonic anhydrase gene (CA), responsible for the conversion between CO_2_ and HCO_3_^−^, was annotated as a key component of CCM [49,50]. The significant increases in g2018 by 26.86-fold suggest urea regulates CCM at the RNA level (Table 3). This implies that urea addition enhances carbon-concentrating mechanisms in microalgae, involving organic carbon in the metabolic processes that promote biological growth. Similar conclusions were drawn in *Microcystis aeruginosa* [51]. Unfortunately, we did not annotate candidate genes responsible for bicarbonate transport, another crucial component of CCM. However, other transporters that may be involved in bicarbonate transport change under nitrogen deficiency conditions and change further under urea conditions [52]. The bile acid: sodium cotransporter (BASS) is thought to mediate the entry of acetoacetic acid into the peroxisome. After nitrogen deficiency, transcription levels of *BASS* gradually increased [53]. Although no differential expression of *BASS* was detected, the sodium/hydrogen exchange family protein (*NDH*: g10029) (Table 3), which is responsible for maintaining the equilibrium of sodium ion inflow, was found to be downregulated by a fold change of 2.64. In addition, we observed upregulated expression of the ATP/ADP transporter (*AAT:* g1797) (Table 3).

Secondly, in terms of photosynthetic carbon sequestration, we pay special attention to genes participating in the Calvin cycle. Those genes were observed, including *PGK* (phosphoglycerate kinase), *GPDH* (glyceraldehyde-3-phosphate dehydrogenase), *RPE* (ribulose-phosphate 3-epimerase), and *TL* (transketolase), showed varying degrees of upregulation (Figure 6 and Table 3). This suggests increased carbon fixation in microalgae, leading to the accumulation of carbon compounds. This signifies the strengthening of the Calvin cycle. The upregulation of genes related to the electron transport chain, associated with the degradation of photosynthetic pigments, combined with urea addition, suggests compensation for the energy consumption caused by pigment degradation, enhancing the Calvin cycle and promoting the carbon fixation capacity of microalgae. Based on physiological measurement results, the total amount of carbohydrates in the experimental group was higher than that in the control group, providing some support for this inference (Figure S2). The key product of the Calvin cycle, glyceraldehyde-3-phosphate (G3P), is transformed into pyruvate through the glycolytic pathway, serving as a precursor in lipid biosynthesis [17].

Thirdly, several genes related to the glycolytic pathway also have different expressions. The downregulation of fructose-1,6-bisphosphate aldolase (*FBPA*: g1829) by 3.02-fold indicates reduced glycolytic activity, emphasizing the accumulation of organic compounds in *N. oceanica* (Figure 6 and Table 3) [54]. In the transcriptome analysis of glycolysis, *ALDO* (aldolase) showed significant downregulation, while *TPI* (triose phosphate isomerase) and *PGK* exhibited significant upregulation (Figure 6 and Table 3). Aldolase plays a crucial role in catalyzing the fourth step of glycolysis, breaking down fructose-1,6-bisphosphate into dihydroxyacetone phosphate (DHAP) and glyceraldehyde-3-phosphate (G3P). TPI facilitates the interconversion between DHAP and G3P, while PGK catalyzes the mutual conversion of 1,3-bisphosphoglycerate and G3P. The differential expression of these three important genes leads to the enrichment of DHAP, providing enhanced energy supply and serving as a key precursor in lipid synthesis, particularly for triacylglycerols (TAGs). Additionally, DHAP participates in lipid degradation through fatty acid beta-oxidation [55].

Fourthly, in the tricarboxylic acid cycle (TCA), the transcription levels of *SDH* (g1987), *FHD* (g8597), and *MDH* (g9301) were upregulated by two-fold (Figure 6 and Table 3), indicating the enhancement of the TCA cycle as a central hub for carbohydrate, lipid, and protein metabolism. On the one hand, it provides an energy supply, and on the other hand, it facilitates the transfer of carbon and nitrogen in response to urea addition. Acetyl-CoA can directly supply the TCA cycle or undergo a bypass process in citrate synthesis using aconitase [56]. Nitrogen concentration and the presence of organic C guide metabolism toward lipid or carbohydrate production [57,58,59]. This indicates that microalgae have adjusted their metabolic strategies. We observed the expression of malate dehydrogenase (*MDH*) in the experimental group, while its FPKM value was 0 in the control group. MDH catalyzes the oxidation of citrate to oxaloacetate, and the produced NADH further contributes to the generation of ATP from ADP, serving as a major source of cellular energy.

Lastly, in the transcriptome analysis of the C4 cycle pathway, only one malic acid dehydrogenase (g9301) was found (Table 3), catalyzing the oxidation of malic acid to oxaloacetate, reducing NAD^+^ to NADH in a reversible reaction. As this enzyme does not participate in CO_2_ fixation, it is more likely involved in supplementing intermediates of the TCA cycle to lipid biosynthesis (Figure 6) rather than redirecting CO_2_ to the plastid for carbon fixation. Acetyl-CoA can also be produced from pyruvate through the “pyruvate dehydrogenase bypass” process, where pyruvate decarboxylase (PDC) and aldehyde dehydrogenase (ALDH) converts pyruvate to acetyl acetate. Although *PDC* was not detected, two ALDH enzymes (g2887, g956) were found (Figure 6 and Table 3). The transcription level of g2887 was upregulated by 2.71-fold, while g956 was downregulated by 2.36-fold. One of the ALDH enzymes (g2887) is located in the cytoplasm, suggesting its involvement in converting cytoplasmic pyruvate (possibly from glycolysis) to acetyl acetate. Acetyl acetate is generated in the cytoplasm through the PDHC pathway and in the mitochondria then forms flexible acetyl-CoA through acetyl-CoA synthetase. These changes may represent a cellular adjustment to energy balance in response to the new growth conditions (urea addition).

### 3.6. Lipid Metabolism Affected by Urea Addition

To investigate the effect of urea addition in *N. oceanica*, our study focused on examining how it influences the fatty acid biosynthetic pathway and the synthesis of triacylglycerol (TAG) in *N. oceanica*. We observed a significant downregulation (2.26-fold) of acetyl-CoA carboxylase (*ACC*) transcript abundance (Figure 6 and Dataset S1). ACC is a crucial enzyme in the first step of fatty acid biosynthesis. Its main function is to convert acetyl-CoA into malonyl-CoA, a key precursor required for fatty acid synthesis. The downregulation of *ACC* results in reduced availability of malonyl-CoA, leading to a decrease in the rate of fatty acid synthesis [17]. Other genes related to fatty acid biosynthesis have not been found in the transcriptome or are not differentially expressed.

For TAG (triacylglycerol), first of all, the synthesis process of TAG involves several enzymatic steps. Glycerol-3-phosphate acyltransferase (GPAT) is the enzyme responsible for the first step in TAG synthesis. It catalyzes the formation of glycerol-3-phosphate fatty acid esters by combining glycerol-3-phosphate and fatty acids. The second step of TAG synthesis is the conversion of glycerol-3-phosphate fatty acid ester into lysophosphatidic acid (LPA), the precursor of TAG, catalyzed by lysophosphatidic acid acyltransferase (LPAAT). Fatty acid synthase (FAS) plays an important role in TAG synthesis of long-chain fatty acids. Fatty acid desaturase is involved in the desaturation of fatty acids by introducing double bonds into the fatty acid chain. However, no differential expression of *GPAT*, *LPAAT*, or *FAS* was found. Nevertheless, the transcriptome analysis revealed the presence of the alpha/beta hydrolase fold protein (g20) (Dataset S1). This protein not only participates in the esterification process of fatty acids but also catalyzes the combination of fatty acids and glycerol to form triglycerides (TAG). Moreover, these proteins also exhibit lipid-soluble activity, breaking down lipid molecules (including TAG) into glycerol and fatty acids. The upregulation of g20 in mRNA expression by 2.40-fold suggests an increased expression of these alpha/beta hydrolase fold proteins under the study conditions. This upregulation may indicate enhanced TAG synthesis and lipolytic activity, potentially influencing energy generation and other metabolic pathways [60,61]. In addition, we found that many other genes for unsaturated fatty acid enzymes are very active. The gene Patatin-like phospholipase, putative (g6254) exhibited a significant upregulation, with its expression level increasing by 3.65-fold (Dataset S1). Similarly, the gene hydrolase, alpha/beta fold family protein (g8602) also showed an upregulation, with its expression level increasing by 2.42-fold (Dataset S1). The putative delta-5 fatty acid desaturase (g1001) and delta-4 fatty acid desaturase (g1678) both exhibit a significant upregulation of over three-fold (Dataset S1). These enzymes are responsible for introducing double bonds by desaturating the saturated carbon chains in fatty acid molecules. This desaturation process contributes to the synthesis of long-chain fatty acids. The downregulation of Alpha/beta hydrolase (g4532) by 2.30-fold suggests a potential inhibition of lipid hydrolysis reactions (Dataset S1). This downregulation could lead to the accumulation of lipids within the system. The common end products of de novo FA biosynthesis are C16:0, C18:0, and C18:1 in the plastids of algae and vascular plants [17].

On the other hand, the metabolic performance of fatty acids is also very important. Transcriptome analysis showed that the enzymes involved in β-oxidation were significantly upregulated. Beta-oxidation (β-oxidation) typically occurs in the mitochondria and is utilized to break down fatty acids to generate energy rapidly. It gradually breaks down long-chain fatty acids into shorter acyl-CoA molecules while simultaneously producing abundant ATP energy. Transcriptome analysis indicates that Acyl-CoA dehydrogenase (g1094) and Acyl-CoA oxidase (g3898) were upregulated by 3.76- and 2.30-fold, respectively (Dataset S1). These enzymes play crucial roles in fatty acid metabolism and are involved in various biochemical processes within the cell. Acyl-CoA dehydrogenase is responsible for catalyzing the alpha, and beta-dehydrogenation of acyl-CoA molecules, a key step in fatty acid degradation. On the other hand, Acyl-CoA oxidase is involved in the oxidation of acyl-CoA molecules. The upregulation of these enzyme genes suggests an increased demand for fatty acid metabolism or energy production in the analyzed context. These enzymes are essential for breaking down fatty acids, which can be used as an energy source or as building blocks for various cellular processes [62]. The upregulation of genes encoded Acyl-CoA dehydrogenase and Acyl-CoA oxidase indicates an enhanced capacity for fatty acid utilization and suggests an active metabolic state in the studied system.

In comparison with other algae studies, it can be found that the response strategies adopted by *N. oceanica* are different. In the presence of urea, *diatoms* tend to exhibit higher lipid synthesis activity. The lipid metabolism strategy of diatoms may favor carbon storage [63]. On the other hand, green algae, in urea conditions, tend to show lower lipid synthesis activity. Green algae may prioritize nitrogen utilization to support growth and protein synthesis [64]. This suggests that green algae may focus more on nitrogen uptake and metabolism rather than lipid accumulation. Based on the measured total lipid content comparison, the experimental group and the control group showed no significant differences, which supports this viewpoint to some extent (Figure S2). Through the analysis of metabolic pathways, it is evident that the proportion of mRNA expression related to unsaturated fatty acids significantly increases. This indicates that under the addition of urea, *N. oceanica* enhances the rate of synthesizing unsaturated fatty acids to adapt to environmental changes. Overall, the addition of urea enhances lipid metabolism. Urea metabolism releases bicarbonate ions (HCO_3_^−^), further promoting the growth and lipid metabolism of *N. oceanica*. Additionally, there are alterations in the composition of lipid production in *N. oceanica*.

### 3.7. Folate Metabolism Affected by Urea Addition

**Figure 7:**
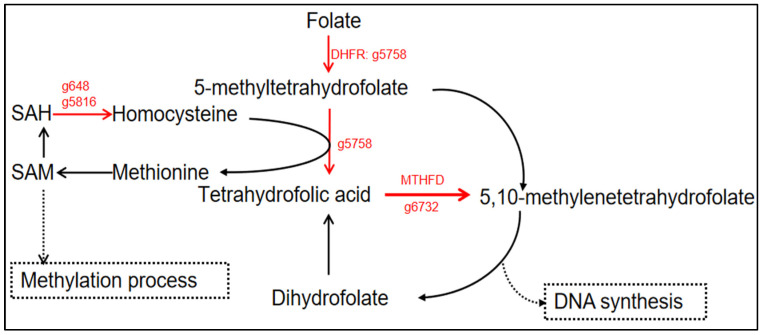
Differential expression analysis of genes related to folate metabolism affected by urea addition in *N. oceanica*. The folate metabolism pathway was briefly constructed. The “red arrows” represent the upregulation of the urea addition condition.

Interestingly, we note a significant activity in the derivative folate metabolism. Folate serves as the predominant supplier and receptor of one-carbon units in the majority of organisms, playing a vital role in various essential metabolic processes such as methylation cycles, amino acid metabolism, and the biosynthesis of nucleotides [65]. The first stage of folate metabolism is commonly facilitated by dihydrofolate reductase (*DHFR*: g5758), employing NADPH as a coenzyme to enzymatically reduce folate to dihydrofolate. Subsequently, DHFR further reduces dihydrofolate to tetrahydrofolic acid (Figure 7 and Dataset S1). Methylenetetrahydrofolate reductase (*MTHFR*: g6732) exhibited a 2.03-fold upregulation at the mRNA level (Figure 7 and Dataset S1). This enzyme, a key regulator in the methyl cycle, catalyzes the conversion of 5,10-methylenetetrahydrofolate (5,10-MTHF) to 5-methyltetrahydrofolate (5-MTHF). It plays a crucial role in folate metabolism, DNA synthesis, and methylation reactions [66]. Adenosylhomocysteinase (g5816) and Methylthioadenosine/S-Adenosylhomocysteine nucleosidase (g648) catalyzes the reversible hydrolysis of S-Adenosylhomocysteine (SAH), breaking it down into adenosine and homocysteine. At the mRNA level, both showed a significant upregulation (5.56-fold and 2.29-fold, respectively) (Figure 7 and Dataset S1). Homocysteine undergoes two metabolic pathways in the body. Firstly, it can be converted into methionine through a remethylation process, involving a key gene for methionine synthesis. Notably, the formation of methionine is associated with remethylation, but the specific gene responsible for the synthesis of methionine has not been detected. Secondly, homocysteine enters the transsulfuration pathway, where it combines with serine catalyzed by cystathionine beta-synthase (*CBS*: g8386, downregulation, FPKM = 0) and its cofactor vitamin B6 (not yet identified) (Dataset S1). This interaction leads to a two-step reaction resulting in the formation of cysteine and α-ketobutyric acid. Seipiapterin Reductase (g1887), an essential enzyme in folate resynthesis, is downregulated by 2.79-fold at the transcriptional level under Ct vs. U (Dataset S1). Folate metabolism likely acts as a pivotal hub, contributing to the redistribution of carbon and nitrogen. On one hand, it plays a role in amino acid synthesis (such as methionine), protein synthesis, DNA methylation, and other biological processes. On the other hand, it may impact gene expression by participating in de novo synthesis of purines and pyrimidines [67,68,69,70].

## 4. Conclusions

Using the mRNA-Seq, we systematically investigated the response of *N. oceanica* to the addition of urea. This study provides novel insights into the regulation mechanism in response to urea addition. Over a 14-day growth period, there was no significant difference in cell concentration in the experimental group compared to the control group. However, at the transcriptional level, *N. oceanica* actively and dynamically regulates the redistribution of carbon and nitrogen after urea addition. To acclimate to urea addition, photosynthesis, carbon/nitrogen metabolism, fatty acid biosynthesis, and lipid metabolism were reprogrammed. Those processes such as amino acid and protein synthesis and degradation, as well as carbon fixation, are enhanced under urea addition. Notably, genes associated with lipases or polyunsaturated fatty acid enzymes were highly activated. These data will serve as a reference for future in-depth research into the effects of urea on *N. oceanica*.

## Figures and Tables

**Table 1:** Differential gene expression involved in nitrogen metabolism in *N. oceanica*.

Gene ID	Gene Name	Abbreviation	Fold Change (U vs. Ct; Fold)
g9735	Kynureninase	KYN	↑ 177.17
g8147	Asparagine synthase	AS	↑ 63.69
g10184	Delta-1-pyrroline-5-carboxylate synthetase	PCS	↑ 6.46
g4389	Similar to dimethylanaline monooxygenase-like (predicted)	DMO	↑ 4.23
g615	Urate oxidase	UOX	↑ 2.12
g8006	Urease accessory protein	URE	↓ 2.17
g6250	Putative urate catabolism protein	UCP	↓ 2.25
g7989	Nitrate high affinity transporter	NAT	↓ 9.35
g6410	Urea/Na+ high-affinity symporter	US	↓ 13.33
g3438	Ferredoxin nitrite reductase	NiR	↓ 17.54
g7791	Ammonium transporter	AMT	↓ 21.7
g7797	Nitroreductase-like protein	NR	↓ 28.57

The symbols “↑” and “↓” represented up- and downregulation, respectively.

**Table 2:** Differential gene expression related to photosynthesis in *N. oceanica*.

Gene ID	Gene Name	Abbreviation	Fold Change (U vs. Ct; Fold)
g5017	Ribulose-phosphate 3-epimerase	RPE	↑ 20.97
g9470	Cytochrome b6-f complex iron–sulfur subunit	PetC	↑ 2.42
g876	Light-dependent protochlorophyllide reductase	LPOR	↑ 2.40
g4344	Iron–sulfur assembly-like protein	ISU	↑ 15.12
g240	Light harvesting complex protein	LHC	↓ 2.04
g5628	Light harvesting complex protein	LHC	↓ 2.05
g7977	3,8-divinyl protochlorophyllide a 8-vinyl reductase, putative chloroplast precursor	DVR	↓ 2.12
g4337	Cytochrome c biogenesis protein, thiol reduction transmembrane region	CcdA	↓ 2.14
g5629	Light harvesting complex protein	LHC	↓ 2.18
g3077	Light harvesting complex protein	LHC	↓ 2.00
g6113	Light harvesting complex protein	LHC	↓ 2.32
g9713	Light harvesting complex protein	LHC	↓ 2.46
g903	Light harvesting complex protein	LHC	↓ 2.55
g5529	Ferredoxin(cyanobacterial type ferredoxin family)	Fd	↓ 6.62

The symbols “↑” and “↓” represented up- and downregulation, respectively.

**Table 3:** Differential gene expression participated in carbon metabolism in *N. oceanica*.

Gene ID	Gene Name	Abbreviation	Fold Change (U vs. Ct; Fold)
**Calvin Cycle**			
g6144	Phosphoglycerate kinase	PGK	↑ 2.18
g10356	Glyceraldehyde-3-phosphate dehydrogenase	GPDH	↑ 2.47
g1829	Fructose-1,6-bisphosphate aldolase	FBPA	↓ 3.02
g8036	Transketolase	TL	↑ 2.07
g5017	Ribulose-phosphate 3-epimerase	RPE	↑ 20.97/3.41
**CCM**			
g2018	Carbonic anhydrase	CA	↑ 26.86/3.06
**C4-like pathway**			
g9301	Malate dehydrogenase	MDH	↑ 265.43
**Degradation of 1,3-β glucan**			
g4700	Glucan 1,3-beta-glucosidase	GluB	↑ 16.24
g5401	Endoglucanase A	EG	↓ 2.07
**Glycolysis**			
g4700	Triosephosphate isomerase	TPI	↑ 2.47
g5401	Phosphoglycerate kinase	PGK	↑ 2.18
g1829	Fructose-1,6-bisphosphate aldolase	ALDO	↓ 3.02
**PDHC Bypass**			
g2887	Aldehyde dehydrogenase	ALDH	↑ 2.71
g956		ALDH	↓ 2.36
**TCA cycle**			
g1987	Succinate dehydrogenase	SDH	↑ 2.01
g8597	Fumarate hydratase	FHD	↑ 2.47
g9301	Malate dehydrogenase	MDH	↑ 265.43
**Transporter**			
g1797	ATP/ADP transporter	AAT	↑ 4.62
g10029	Sodium/hydrogen exchanger	NDH	↓ 2.64

The symbols “↑” and “↓” represented up- and downregulation, respectively.

## Data Availability

The original contributions presented in the study are included in the Supplementary Materials, further inquiries can be directed to the corresponding author.

## Supplementary Materials

The following supporting information can be downloaded at: https://www.mdpi.com/article/10.3390/life14070797/s1, Dataset S1: The mRNA-Seq data for the genes involved in glycerolipid metabolism, fatty acid biosynthesis, lipases, carbon fixation and CCM, carbon metabolism, and transporter, nitrogen metabolism, folate metabolism, and beta-oxidation. Table S1: Real-time PCR primer sequences for the six genes used in real-time PCR experiments to validate the mRNA-Seq results. Figure S1: RT-qPCR analysis. Figure S2: Measurement of carbohydrate, protein and total lipid. Figure S3. Measurement of photosynthetic parameter Fv/Fm.
