# Supplementary material for: Transcriptomic Analysis Reveals the Effect of Urea on Metabolism of Nannochloropsis oceanica
Source: Life (Basel). 2024 Jun 24;14(7):797. doi: 10.3390/life14070797 (PMC11278182; doi:10.3390/life14070797)
Supplement: Supplementary file 1 [file life-14-00797-s001.zip › Figures.pdf]

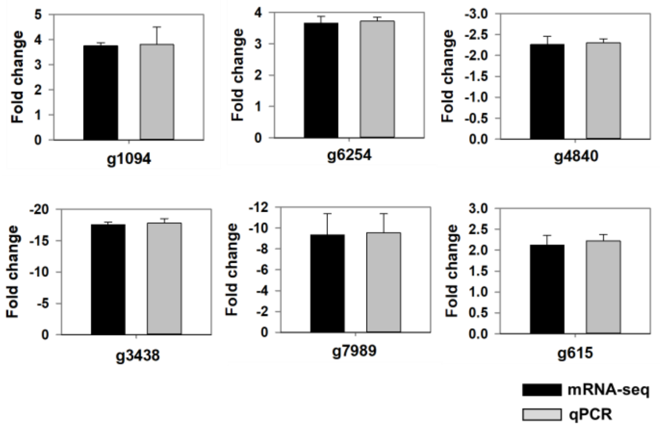

Figure S1. RT-qPCR analysis.

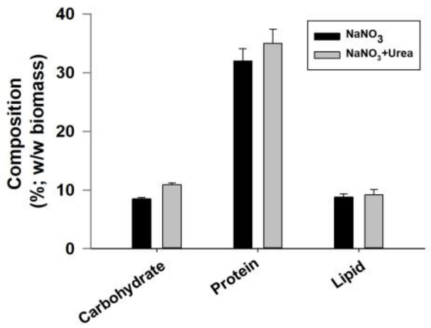

Figure S2. Measurement of carbohydrate, protein and total lipid.

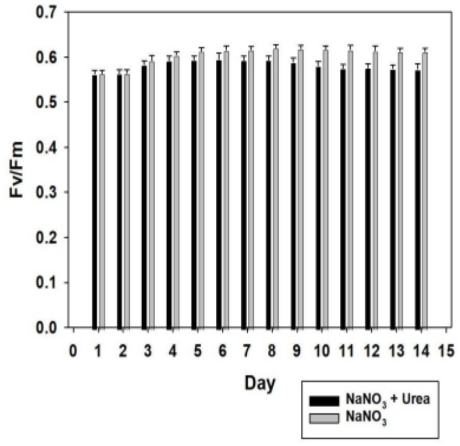

Figure S3. Measurement of photosynthetic parameter Fv/Fm.
