# Supplementary material for: Transcriptomic Analysis Reveals the Effect of Urea on Metabolism of Nannochloropsis oceanica
Source: Life (Basel). 2024 Jun 24;14(7):797. doi: 10.3390/life14070797 (PMC11278182; doi:10.3390/life14070797)
Supplement: Supplementary file 1 [file life-14-00797-s001.zip › Table S1_qPCR primers.pdf]

Supplemental Table 1. Real-time PCR primer sequences for the six genes used in real-time PCR experiments to validate the mRNA-Seq results.

Gene Name	Gene ID	Forward Primer	Reverse Primer
**Actin**	g3056	5'GCCGTTATTGGATGGATATG3'	5'ACAACAACTCTCCTTCACA3'
**Acyl-CoA dehydrogenase**	g1094	5'AGTTCTTCCAGGCAGCTTAC3'	5'TCACCGGGGCCCAGAGAGT3'
**Patatin-like phospholipase**	g6254	5'AGCCCGTGGGACCCCTCAGT3'	5'CCAACCGACAGCCGACCGTT3'
**acetyl-CoA carboxylase**	g4840	5'TATATCTATCTAGGACGAAGGC3'	5'ATGGTGGTGGCCATTATTGT3'
**Urate oxidase**	g615	5'GATACCCTGATTGATGAATTTG3'	5'CAAAGATCTCTGACCTCCAC3'
**Nitrate high affinity transporter**	g7989	5'AACCTGATACTGCGCATCAT3'	5'TCTTGAACATGTTGGTGGTC3'
**Ferredoxin nitrite reductase**	g3438	5'AAAGCTTAAGAAGGACGGCT3'	5'GAAGCTGAAGGTTTTGCC3'
